# Fatal, Fulminant, Necrotizing Pancreatitis Associated With Recent Tirzepatide Initiation

**DOI:** 10.1210/jcemcr/luaf087

**Published:** 2025-04-23

**Authors:** Krista Grennan, Courtney Borg, Ashley Meneley, Tyler Janitz, Midiia Shuman, Carla Venegas

**Affiliations:** Department of Internal Medicine, Mayo Clinic, Jacksonville, FL 32224, USA; Department of Anesthesiology, Mayo Clinic, Jacksonville, FL 32224, USA; Department of Neurology, Mayo Clinic, Jacksonville, FL 32224, USA; Department of Family Medicine, Mayo Clinic, Jacksonville, FL 32224, USA; Department of Critical Care Medicine, Mayo Clinic, Jacksonville, FL 32224, USA; Department of Critical Care Medicine, Mayo Clinic, Jacksonville, FL 32224, USA

**Keywords:** weight loss, obesity, tirzepatide, pancreatitis

## Abstract

As obesity is increasing in prevalence and its associated morbidity and mortality are escalating worldwide, medications like tirzepatide are frequently used for weight loss benefits in patients without diabetes. Tirzepatide has rarely been associated with acute pancreatitis, but there are no reported cases of fulminant, necrotizing pancreatitis resulting in fatality. We present a case of fatal, fulminant, necrotizing pancreatitis in a 64-year-old low-risk female patient with recent tirzepatide initiation. Prior to starting this medication, a case-by-case risk benefit analysis should be performed for each patient. The importance of this should be emphasized by making necrotizing pancreatitis a boxed warning for tirzepatide.

## Introduction

The prevalence of obesity in American adults is expected to exceed 50% by 2030 [1]. This will likely be paralleled with an increase in associated cardiovascular risk factors and reduced life expectancy. Tirzepatide, a dual glucose-dependent insulinotropic polypeptide and glucagon-like peptide-1 (GLP-1) receptor agonist, is currently approved for use in patients with type 2 diabetes mellitus to regulate glucose metabolism and insulin secretion. It is also approved and often used for its significant weight loss benefits in patients with obesity without diabetes [2]. Tirzepatide is known to rarely cause acute pancreatitis in patients with its incidence being approximately 0.39% [3]. Proposed mechanisms of GLP-1 receptor agonists contributing to acute pancreatitis include the stimulation of GLP-1 receptors in pancreatic islet β cells and exocrine duct cells. This potentially causes an overgrowth of the cells covering the smaller ducts, leading to occlusion and buildup of pancreatic enzymes with subsequent acute pancreatic inflammation [4]. If the inflammation and damage is severe enough, the pancreas can develop necrosis. There have been no specific case reports documenting fulminant, necrotizing pancreatitis resulting in fatality with tirzepatide use. Deaths associated with adverse effects from tirzepatide (which include severe hypoglycemia, acute pancreatitis, cholelithiasis, and cholecystitis) are estimated at ≤1% regardless of the dose [3]. Here we present a case of fatal, fulminant, necrotizing pancreatitis in a patient with recent tirzepatide initiation.

## Case Presentation

A 64-year-old female with hyperlipidemia and class 1 obesity (height 167.6 cm, weight 86.2 kg, body mass index 30.67 kg/m^2^) presented to the emergency department with sudden onset epigastric abdominal pain, diaphoresis, diarrhea, and chills for the past 7 hours. The patient had recently initiated tirzepatide 2.5 mg weekly for weight loss with no history of type 2 diabetes mellitus. Her most recent dose of 2.5 mg weekly was administered 4 days prior to admission. The patient estimated that she received 2 to 4 doses total of the medication. She reported alcohol use in the form of approximately 1 to 2 glasses of wine, 3 times per week. She had no history of binge drinking or alcohol use disorder. There was no recent abdominal trauma. She had no history of gallbladder disease, gallstones, biliary disease, or acute or chronic pancreatitis. Her only other medications were simvastatin 20 mg daily for hyperlipidemia and escitalopram 10 mg daily for depression. Both of her chronic medications had no recent dose adjustments.

## Diagnostic Assessment

At the time of presentation, the patient was afebrile with a normal heart rate (98 beats per minute), tachypnea (22 breaths per minute), hypertension (167/66 mmHg), and oxygen saturation of 96% on room air. Her physical exam was remarkable for diffuse upper abdominal pain with guarding.

On admission, the patient was found to have a lipase of 8195 U/L [137 µkat/L] (normal reference range: 13-60 U/L; 0-2.67 µkat/L), aspartate aminotransferase of 30 U/L [0.5 µkat/L] (normal reference range: 8-43 U/L; 0-0.58 μkat/L), alanine aminotransferase of 32 U/L [0.5 µkat/L] (normal reference range: 7-45 U/L; 0.12-0.50 µkat/L), total bilirubin of 0.4 mg/dL [6.8 μmol/L] (normal reference range: 0.1-1.2 mg/dL; 1.71 to 20.5 µmol/L), glucose of 234 mg/dL [13.0 mmol/L] (normal reference range: 70-140 mg/dL; 3.9-5.5 mmol/L), hemoglobin of 17.7 g/dL [11.0 mmol/L] (normal reference range: 11.6-15.0 g/dL; 8.4-10.2 mmol/L), hematocrit of 53.8% (normal reference range: 35.5-44.9%), triglycerides of 147 mg/dL [1.7 mmol/L] (normal reference range: < 150 mg/dL; < 1.7 mmol/L), and a white blood cell count of 18.9 × 10^9^/L [18.9 K/µL] (normal reference range: 3.4-9.6 × 10^9^; 4.5-11 K/µL). Urinalysis was negative for signs of infection.

**Figure 1.**
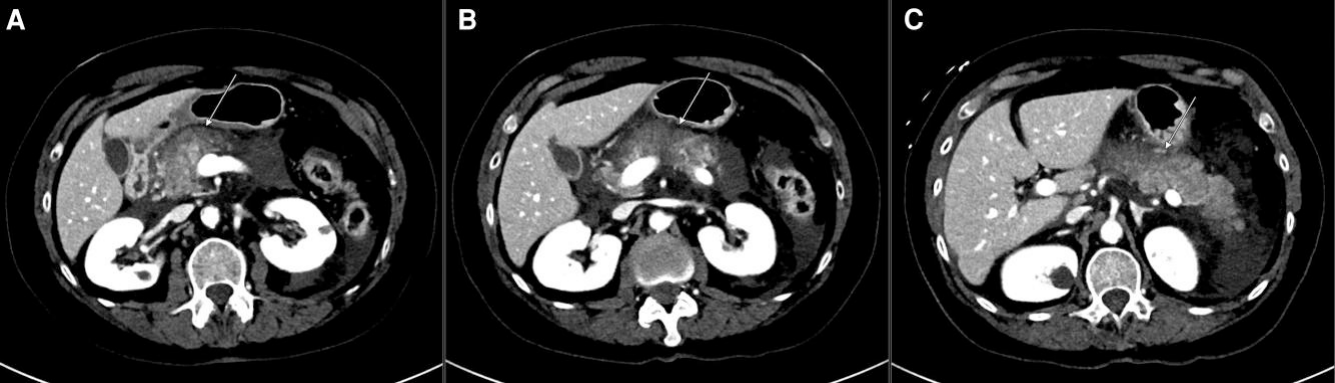
(A-C) Computed tomography scan of the abdomen and pelvis showing heterogeneous enhancement of the pancreatic body with areas of significantly decreased enhancement suggestive of acute pancreatitis with probable partial necrosis of the pancreatic body. There is associated peripancreatic and bilateral paracolic fluid. Arrows pointing to the pancreas.

Initial computerized tomography (CT) scan of the abdomen and pelvis revealed peripancreatic, paracolic, and free pelvic fluid with no pancreatic duct dilation or calcification consistent with acute pancreatitis (Fig. 1A-1C). There was also evidence of partial possible necrosis of the pancreatic body and hepatic steatosis as demonstrated by low attenuation of the liver. Ranson's criteria score was 3, indicating a 15% predicted mortality [5]. BISAP score was 2 (1 point for 2 or more systemic inflammatory response syndrome criteria and 1 point for age greater than 60) without a chest radiograph on admission to assess for pleural effusion, which limited the utility of the score in prediction of mortality [6]. The patient was admitted for acute pancreatitis and initiated on intravenous (IV) fluids and pain medications.

**Figure 2.**
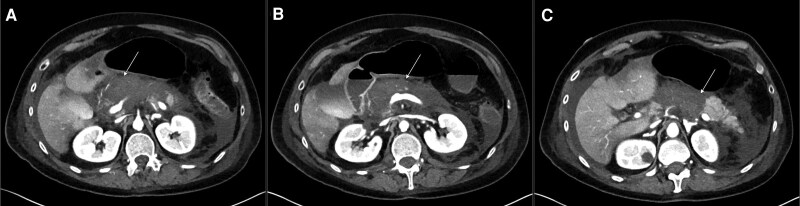
(A-C) Computed tomography scan of the abdomen and pelvis demonstrating absence of parenchymal enhancement in the pancreatic body, pancreatic neck, and a portion of the pancreatic head, suggestive of worsening necrotizing pancreatitis. Increased peripancreatic inflammatory changes and ill-defined peripancreatic fluid. Arrows pointing to the pancreas.

On day 2 of the hospital admission, the patient had persistent severe upper abdominal pain and was not tolerating oral feeds. Repeat CT of the abdomen and pelvis showed interval worsening of severe necrotizing pancreatitis with necrosis noted in the pancreatic body, neck, and part of the head (Fig. 2A-2C). There was also evidence of hypoperfusion including heterogeneous enhancement of the liver with hyperenhancement of kidneys, adrenal glands, and small intestine. CT of the chest showed bilateral small to moderate pleural effusions (left greater than right) with diffuse ground glass opacities and bibasilar atelectasis. Morning laboratory studies on day 2 revealed a new transaminitis with aspartate aminotransferase of 2194 U/L [36.6 µkat/L] and alanine aminotransferase of 1559 U/L [26.0 µkat/L].

## Treatment

In the afternoon of day 2 of hospital admission, the patient began developing significant respiratory distress and was admitted to the intensive care unit where she was subsequently intubated and placed on a mechanical ventilator due to acute hypoxemic respiratory failure. She developed distributive shock requiring norepinephrine, vasopressin, phenylephrine, and angiotensin II IV infusions for blood pressure support.

Follow-up evening labs on day 2 of admission showed metabolic acidosis with pH of 7.264 (normal reference range: 7.350-7.450), normal pCO_2_, and a bicarbonate of 19.7 mEq/L [19.7 mmol/L] (normal reference range: 22.0-26.0 mEq/L; 22.0-26.0 mmol/L), hypocalcemia with a total corrected calcium of 7.5 mg/dL [1.9 mmol/L] (normal reference range: 8.8-10.2 mg/dL; 2.2 to 2.7 mmol/L) and ionized calcium of 3.6 mg/dL [0.9 mmol/L] (normal reference range: 4.7-5.4 mg/dL; 1.1-1.3 mmol/L), anemia with a hemoglobin of 9.7 g/dL [6.0 mmol/L] and hematocrit of 30.1%, and thrombocytopenia with a platelet count of 81000 cells/µL [81 × 10^9^/L] (normal reference range: 150000-400000 cells/µL; 157-371 × 10^9^/L), which decreased from 280000 cells/µL [280 × 10^9^/L] on admission.

There was concern for disseminated intravascular coagulation; thus, the patient was administered packed red blood cells and platelets. Her lactate was 12.3 mEq/L [12.3 mmol/L] (normal reference range: 0.5-2.2 mEq/L; 0.5-2.2 mmol/L). The patient's urine culture also was positive for *Enterococcus faecalis,* so she was initiated on amikacin, meropenem, and vancomycin. Furthermore, the patient was oliguric and initiated on continuous renal replacement therapy.

## Outcome and Follow-up

On day 3 of hospital admission, labs were suggestive of disseminated intravascular coagulation with prothrombin time of 114.7 seconds (normal reference range: 9.4-12.5 seconds), international normalized ratio of 9.9 (0.9-1.1), activated partial thromboplastin time of 76 seconds (normal reference range: 25-37 seconds), fibrinogen of 94 mg/dL [2.8 µmol/L] (normal reference range: 200-393 mg/dL; 5.9-11.6 µmol/L), and d-dimer of 12496 ng FEU/mL [12.5 mg FEU/L] (normal reference range: less than or equal to 500 ng FEU/mL; less than or equal to 0.5 mg FEU/L).

On exam, the patient was noted to have increased abdominal distention, elevated intra-abdominal pressure, and asymmetric pupils. Stroke evaluation protocol was initiated, but the patient developed asystole enroute to the CT scanner. No resuscitation was performed due to the patient's do not resuscitate status, and she was pronounced as decreased. Autopsy results showed necrotic pancreas with surrounding fat necrosis.

## Discussion

This is the only case of fatal, necrotizing pancreatitis in a patient on tirzepatide documented in the literature. While tirzepatide has known significant weight loss benefits, physicians should be aware of the rare but potentially fatal side effects. A case-by-case risk benefit analysis should be performed in conjunction with shared decision-making prior to initiating this medication. While adverse effects are rare, they may result in fatalities [3]. Physicians should have significant discussions with their patients about the rare but potentially fatal side effects associated with the glucose-dependent insulinotropic polypeptide and GLP-1 receptor agonist class of medications.

Physicians should also be cautious when prescribing and adjusting the dose of tirzepatide to minimize the risk of pancreatitis and other adverse effects. Prior to starting the medication, physicians should screen patients for a pancreatitis history as this may be a relative contraindication to a patient starting the medication depending on the circumstances of the patient's previous pancreatitis episodes. Additionally, it is recommended that the dose of tirzepatide be increased at intervals of 2.5 mg after at least 4 weeks at the current dose to minimize the risk of all side effects including pancreatitis [7].

Additionally, lifestyle modifications should be attempted before considering tirzepatide use. There is extensive literature from the Diabetes Prevention Program and the Diabetes Prevention Program Outcomes Study that support intensive lifestyle interventions having positive effects on overall health outcomes [8, 9]. Patients with intensive lifestyle interventions and those exclusively on metformin saw a 27% and 18% lower incidence of developing diabetes over the 15-year study period when compared with the placebo group, despite weight regain in both groups [9]. Additionally, the lifestyle interventions group saw a significant reduction in microvascular complications of diabetes compared to the placebo group in patients who did not go on to develop diabetes [9]. Both the lifestyle interventions group and metformin group had no increased risk of adverse effects compared to placebo [9]. These studies highlight the benefits of intensive lifestyle interventions, even without sustained weight loss, on health outcomes [8, 9].

Lifestyle interventions alone do have limitations as many patients who lose weight with intensive lifestyle interventions often regain approximately 50% of the weight that they lose [9, 10]. It also only has a modest reduction in weight of about 5% of total body weight lost after 2 years of intensive diet and exercise [10].

While tirzepatide is known to rarely have serious, potentially fatal side effects, 2 meta-analyses found that tirzepatide appeared to be safe regarding the risk of pancreatitis with no significant increased risk [11, 12]. The 2024 meta-analysis found that tirzepatide does increase pancreatic amylase and lipase more than placebo or insulin; however, the clinical significance of this has not been characterized [12].

Our patient had no other risk factors for pancreatitis except tirzepatide usage. While the patient did report a history of alcohol consumption in the form of 1 to 2 glasses of wine 3 times per week, available data suggest that alcohol-induced pancreatitis is more so associated with beer and spirits (rather than wine), drinking every day, and frequent binge drinking [13].

The significance of this potentially fatal side effect of tirzepatide should be emphasized by making necrotizing pancreatitis a boxed warning for tirzepatide. Additionally, future studies should be aimed at assessing the long-term health effects of tirzepatide including the risk of pancreatitis with direct comparison to lifestyle interventions, metformin, and placebo to help physicians make more informed recommendations for their patients with diabetes and/or obesity.

## Learning Points

- Tirzepatide is a helpful drug for diabetes and weight loss, but its potentially fatal side effects must not be overlooked.
- Lifestyle modifications should be attempted prior to tirzepatide initiation.
- Shared and informed decision-making should be used when considering tirzepatide initiation.

## Contributors

All authors made individual contributions to authorship. K.G., C.B., A.M., T. J., M.S., and C.V. were involved in the diagnosis and management of the patient. K.G. was involved in all sections of the manuscript and submission. C.B. was involved in figure creation and manuscript writing. A.M. was involved in manuscript writing in the case description portions. She also did significant literature review. T.J. was involved in the manuscript discussion section plus the manuscript editing. M.S. was involved in manuscript editing, introduction development, and reference management. C.V. is the senior author responsible for significant edits and literature review. All authors reviewed and approved the final draft.

## Data Availability

Data sharing is not applicable to this article as no datasets were generated or analyzed during the current study.
